# Transcriptomic Analysis of Porcine Granulosa Cells Overexpressing Retinol Binding Protein 4

**DOI:** 10.3390/genes10080615

**Published:** 2019-08-13

**Authors:** Yun Zhao, Chunjin Li, Xu Zhou

**Affiliations:** 1 College of Animal Sciences, Jilin University, Changchun 130062, China; 2 Jilin Provincial Key Laboratory of Animal Embryo Engineering, Jilin University, Changchun 130062, China

**Keywords:** granulosa cells (GCs), high-throughput sequencing, pLVX-IRES-ZsGreen vector, retinol binding protein 4 (*RBP4*)

## Abstract

Retinol binding protein 4 (RBP4), mainly secreted by the liver and adipocytes, is a transporter of vitamin A. RBP4 has been shown to be involved in several pathophysiological processes, such as obesity, insulin resistance, and cardiovascular risk. Reports have indicated the high expression levels of *RBP4* in cystic follicles. However, the role of *RBP4* in mammalian follicular granulosa cells (GCs) remains largely unknown. To illustrate the molecular pathways associated with the effects of *RBP4* on GCs, we used high-throughput sequencing to detect differential gene expression in GCs overexpressing *RBP4*. A total of 113 differentially expressed genes (DEGs) were identified in *RBP4*-overexpressing GCs, and they included 71 upregulated and 42 downregulated genes. The differential expressions of the top 10 DEGs were further confirmed by real-time quantitative polymerase chain reaction. Pathway analysis indicated that the DEGs are mostly involved in oxidative phosphorylation, Parkinson’s disease, non-alcoholic fatty liver disease, Huntington’s disease, cardiac muscle contraction, Alzheimer’s disease, fatty acid biosynthesis, AMP-activated protein kinase signaling pathway, and insulin signaling pathway. Genes in these pathways should be useful for future studies on GCs. Altogether, the results of our study establish a framework for understanding the potential functions of *RBP4* in porcine GCs.

## 1. Introduction

Retinols (a metabolite form of vitamin A, ROH) mediate various reproductive processes, such as follicle development, oocyte maturation, early embryogenesis, and steroidogenesis [1,2,3,4]. Retinol binding protein 4 (RBP4) is an adipokine belonging to the lipocalin family of proteins; it plays a role in the transport of retinols from liver and adipose tissues to the blood and other tissues [5,6]. The pivotal action of RBP4 is mediated by a plasma membrane receptor stimulated by retinoic acid 6 (STRA6) [7]. RBP4 exerts its function by binding retinols in blood to form a transient RBP4-ROH complex. STRA6 facilitates the dissociation of retinols from the complex and transports them into cells [8]. RBP4, which is involved in retinol metabolism, has also been identified to be associated with numerous metabolic diseases, such as insulin resistance (IR), type 2 diabetes mellitus, obesity, and cardiovascular risk [9,10]. *RBP4* expression in adipose tissues is regulated by 17-β-estradiol. RBP4 levels in serum are elevated in polycystic ovary syndrome (PCOS) women with obesity [11]. RBP4 can drive ovarian cancer cell migration and proliferation through RhoA/Rock1 and extracellular signal-regulated kinase pathways involving matrix metalloproteinase (MMP) 2 and MMP9 expressions [12].

The roles of *RBP4* in reproduction have been supported by accumulating studies. A recent study identified a new single nucleotide polymorphism in *RBP4* affecting the production of large litter size of sows [13]. Studies have shown that *RBP4* expresses in bovine ovarian cells [14], early vitellogenic oocytes of the trout ovary [15], and ovaries of zebrafish [16]. In addition, *RBP4* is expressed during the period of pig blastocyst [17], in the gravid endometrium around day 12 of pregnancy of sows [18]. RBP4 has also been found to be a major secretory product of the pig conceptus [19]. Researchers also reported that the most advanced conceptuses secrete the RBP4 necessary for their development [20]. These evidences suggest that *RBP4* plays important roles in ovarian, uterine, and conceptus physiology.

Ovarian granulosa cells (GCs) are important somatic cells and play a central role in steroidogenesis, which is critical for female reproduction. The growth and development of GCs are regulated by a complex cell signaling system, which significantly influences follicular development and oocyte maturation. Factors regulating functions of GCs may therefore play important roles in follicular health and female reproduction [21]. The levels of *RBP4* and retinols in the fluids of large follicles are higher than those in the fluids of medium or small follicles [14]. The expression of *RBP4* can also be detected in ovarian GCs of various species, including pigs [22], mice [23], and cows [14]. Furthermore, our previous studies have detected high concentrations of RBP4 in porcine cystic follicles [22]. A recent study revealed that *RBP4* is highly expressed in follicular fluids of large porcine follicles. RBP4 can also increase the expressions of follicle-stimulating hormone receptor (*FSHR*) and luteinizing hormone receptor (*LHR*) in GCs [24]. These data suggest that ovarian *RBP4* might be involved in normal ovarian function. However, current available information on the function and regulatory mechanism of *RBP4* in GCs is limited. This study aimed to analyze the function of the porcine *RBP4* gene using gene overexpression and high-throughput sequencing.

## 2. Materials and Methods

### 2.1. Obligatory Ethical Approval

All experiments in the present study were performed in accordance with the guidelines of the Animal Care and Use Committee of Jilin University Changchun, China (approval number: JLU#20150903).

### 2.2. Cell Culture

Unless otherwise specified, all the chemicals and reagents were purchased from Takara (Takara, Dalian, China). The 293T cell line (ATCC, CRL-11268; Manassas, VA, USA), which were used to generate lentivirus, were cultured in Dulbecco’s Modified Eagle Medium/Ham’s F-12 (DMEM/ F12) (Hyclone, Logan, UT, USA) supplemented with 10% fetal bovine serum (FBS) (Invitrogen, Carlsbad, CA, USA) and 1% penicillin–streptomycin under humidified atmosphere containing 5% CO_2_ at 37 °C

The current study followed the culture procedure of a previous method [25]. Landrace porcine ovaries were collected from pre-pubertal gilts aged 165–180 days from a local slaughterhouse and transported to laboratory within 20 min in saline at 37 °C. The follicular fluid aspirated from 3–6 mm follicles was centrifuged at 500× *g* for 5 min and washed thrice with phosphate buffer saline (PBS). The viability of cells before cell culture was assessed by trypan blue exclusion assay. The GCs were seeded at an initial density of 1 × 10^6^ cells/mL in six-well plates. The culture medium consisted of DMEM/F12 with 10% FBS and 1% penicillin–streptomycin. The cells were incubated under humidified atmosphere containing 5% CO_2_ at 37 °C for 24 h and then washed with PBS to remove any unattached cells.

### 2.3. Plasmid Constructs, Transfection, and Infection

The primer sequences used for *RBP4* amplification containing XhoI and NotI restriction enzyme sites in the forward and reverse primers included 5′-TTC CTC GAG ACT ATG GAA TGG GTT TGG GCG CTC GTG CT-3′ and 5′-CCG CGG CCG CTC CTA CAA AAT GTT TCT TTC CGA TTT GC-3′. Full-length porcine *RBP4* complementary DNA (cDNA, GenBank Accession No. 397124) was amplified and inserted into the XhoI/NotI sites of the pLVX-IRES-ZsGreen lentiviral vector. *RBP4* polymerase chain reaction (PCR) products and pLVX-IRES-ZsGreen plasmid were excised by digesting with XhoI and NotI respectively. Porcine *RBP4* gene was inserted into the pLVX-IRES-ZsGreen lentiviral vector to generate the pLVX-*RBP4*-IRES-ZsGreen construct. Approximately 24 h before transduction, 293T cells were seeded into 100 mm plates at 4 × 10^6^ cells/plate and 10 mL growth medium and then incubated at 37 °C, 5% CO_2_ overnight. Then, the cells were further incubated until 80–90% confluency. Four packaged systems of plasmids (Invitrogen, Carlsbad, CA, USA) included pLVX-IRES-ZsGreen vector, structure plasmid PLP1, PLP2, and envelope plasmid VSVG. FuGENE HD reagent (Roche, Mannheim, Germany), following the manufacturer’s instructions, was used to transfect the 293T cells by mixing vector plasmid pLVX-*RBP4*-IRES-ZsGreen, structure plasmid PLP1, PLP2, and envelope plasmid VSVG at a mass ratio of 4:2:1:1. The cells with blank pLVX-IRES-ZsGreen (vector) transfection were used as negative controls. After 48 and 72 h, viral particles from the culture supernatants were collected by ultracentrifugation at 4 °C, 72,000× *g* for 2.5 h. A limiting dilution assay established that the titer of the virus detected by the green fluorescent protein (GFP)-labeled method was 1.2 × 10^7^  transduction units (TU)/mL according to previously reported protocol [26]. GCs were divided into two groups: pLVX-*RBP4*-IRES-ZsGreen (pLVX-*RBP4*) and pLVX-IRES-ZsGreen (CTRL) groups. Each group was performed in biological triplicate. The lentiviral particles were added to the GCs at a multiplicity of infection of 50. The GCs were infected with lentiviral particles for 72 h, and the medium with lentiviral particles was replaced every 24 h. After 72 h of incubation, enhanced GFP (EGFP) was observed under a fluorescence microscope. *RBP4* expression was quantified by real-time quantitative polymerase chain reaction (RT-qPCR) and Western blotting analysis.

### 2.4. Total RNA Extraction and Real-Time Quantitative Polymerase Chain Reaction (RT-qPCR)

Total RNA of GCs from three independent biological repetitions of the pLVX-*RBP4* and CTRL groups with TRIZOL reagent (Qiagen, Hilden, Germany) was extracted according to manufacturer’s protocol. RNA concentration and purification were assessed using a NanoDrop 2000 spectrophotometer (Thermo Fisher, Waltham, MA, USA); the number of OD260/OD280 must be higher than 1.8. RNA integrity was detected by agarose gel electrophoresis. Total RNA was reverse-transcribed into cDNA by using PrimeScript 1st strand cDNA Synthesis Kit (Takara). The total volume of 20 µL RT-PCR reaction mixture contained 10 µL SYBR^®^ Green Real-time PCR Master Mix (Takara), 2 µL cDNA, 7 µL ddH_2_O, and 0.5 µM each of forward and reverse primers. The program used for all genes consisted of a denaturing cycle of 120 s at 95 °C, 40 cycles of PCR (94 °C for 30 s, 59–61 °C for 30 s, and 72 °C for 60 s), a melting cycle consisting of 95 °C for 30 s, 72 °C for 5 min, and a step cycle starting at 65 °C with a 0.2 °C/s transition rate to 95 °C. RT-qPCR analysis was conducted on an Mx3000P system (Stratagene, San Diego, CA, USA) with SYBR-Green detection according to manufacturer’s instructions. The expression level of GAPDH was used as endogenous control. Relative expression was calculated using the 2^−ΔΔCt^ method. The primer sequences are listed in Table S1.

### 2.5. Western Blotting

Extraction of proteins from GCs and subsequent quantification were performed as previously described [27]. Equal amounts (40 µg) of proteins were resolved by 10% sodium dodecyl sulfate polyacrylamide gel electrophoresis and transferred onto polyvinylidene fluoride membranes. After blocking, the membranes were incubated overnight at 4 °C with anti-RBP4 mouse monoclonal antibody (1:300; Bioworld, Nanjing, China) and anti-β-actin rabbit monoclonal antibody (1:1000; Boster, Wuhan, China). After incubation with the primary antibodies, the membranes were washed thrice with TBST and then incubated for 1 h with 3000-fold diluted HRP-labeled goat anti-rabbit or goat anti-mouse secondary antibodies (Boster, Wuhan, China) at room temperature. After incubation, the membrane was washed thrice with TBST at 5 min intervals. After washing, each membrane was covered with enhanced chemiluminescence Western blotting reagents (Beyotime, Shanghai, China) for the detection of protein bands.

### 2.6. RNA-Sequencing Library Construction 

Six RNA-sequencing (RNA-seq) libraries (three RNA-seq libraries for pLVX-*RBP4* group and three RNA-seq libraries for CTRL group) were generated using an NEBNext Ultra RNA Library Prep Kit for Illumina (NEB, Ipswich, MA, USA) according to the manufacturer’s recommendations. A total amount of 1 µg RNA per sample was submitted to HiSeqTM 2500 (Illumina Inc., San Diego, CA, USA) for cDNA library preparation. The library was sequenced by pair-end sequencing, and 125 bp paired end reads were generated. Primary sequencing data produced using the Illumina HiSeqTM 2500 system were considered raw reads. Raw sequencing data generally featured clean reads, reads with a sequencing primer, tags containing N, and adapter sequences. Raw reads were filtered into clean reads by FAST-QC [28]. 

### 2.7. Identification of Differentially Expressed Genes (DEGs) 

TopHat2 software was used for aligning the clean reads to the current pig reference genome (Sscrofa10.2) [29]. The unigene numbers were converted into Entrez Gene ID numbers using the online g:profile conversion tool (http://biit.cs.ut.ee/gprofiler/). Gene expression levels were calculated by fragments per kilo bases per million mapped reads (FPKM). The screening of DEGs used Cuffdiff command of Cufflinks Software. The criteria filtering DEGs were as follows: (1) *p*-value < 0.05 and *q*-value (adjusted *p*-value) < 0.05; (2) |fold change| ≥ 2 or ≤1 in both groups. The criteria for screening the top 10 genes are based on fold change values.

### 2.8. Functional Enrichment Analysis

**Figure 1:**
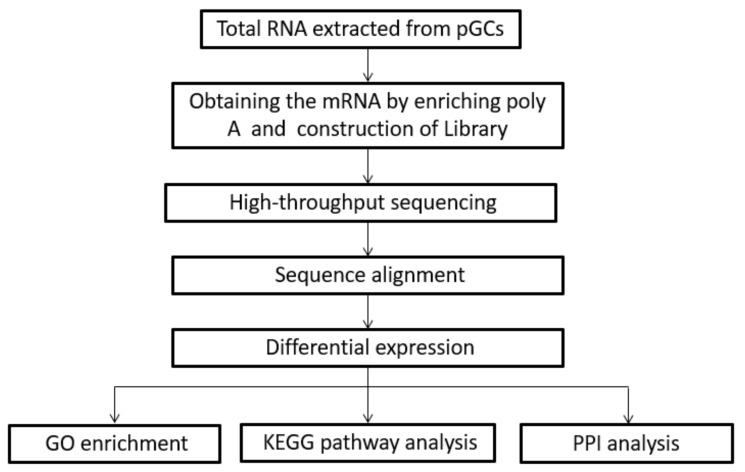
Workflow of high-throughput sequencing. GC: Granulosa cells, GO: gene ontology, KEGG: Kyoto Encyclopedia of Genes and Genomes, PPI: Protein-protein interaction

Go term enrichment analysis was conducted for upregulated and downregulated genes using the R package clusterProfiler [30]. In GO term tests, completely annotated genes of swine were appointed as the background. Kyoto Encyclopedia of Genes and Genomes (KEGG) (http://www.genome.jp/kegg) was used to identify the metabolic pathways or signal transduction pathways that were significantly enriched with DEGs by comparing them to the entire genome background (Figure 1). Fisher’s exact test was applied to identify the significant GO categories and KEGG pathways. Only GO terms or KEGG pathways with *p*-values < 0.05 were considered to be significantly enriched [31]. Protein–protein interaction (PPI) analysis of DEGs was based on the STRING database v11.0 [32]. All RNA-seq data were deposited into the Gene Expression Omnibus (GEO) database from NCBI (https://www.ncbi.nlm.nih.gov/geo). The accession record of GEO is GSE132962.

### 2.9. Statistical Analysis

SPSS version 17.0 (SPSS, Inc., Chicago, IL, USA) was used for statistical analysis. The Student’s *t*-test was performed to analyze statistical differences between means. All values were expressed as mean ± SEM (standard error of the mean). *p* < 0.05 was considered statistically significant. 

## 3. Results

### 3.1. Infection Efficiency

**Figure 2:**
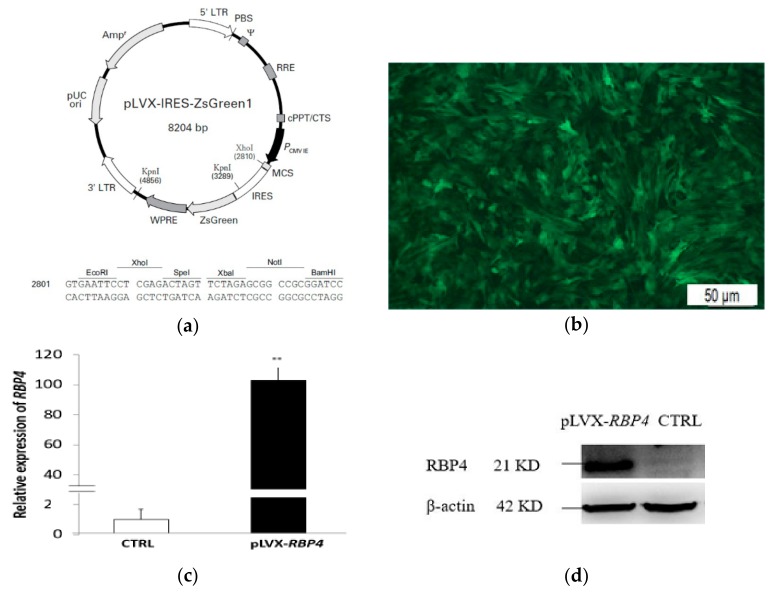
Overexpression of retinol binding protein 4 (*RBP4*) in GCs. (**a**) pLVX-IRES-ZsGreen vector. (**b**) GCs expressing green fluorescent protein (GFP) could be observed after infection by *RBP4* lentivirus particles under fluorescence microscope (20×). (**c**) *RBP4* mRNA expression levels in GCs after 72 h of lentivirus transfection. (** *p* < 0.01). (**d**) RBP4 protein (21 KD) expression upon *RBP4* overexpression in GCs. β-Actin was used as an internal control (42 KD). CTRL: control.

The recombinant lentiviral was successfully infected into GCs. EGFP was detected by fluorescence microscopy in the transfected group (Figure 2a,b). RT-qPCR indicated a significant increase in *RBP4* messenger RNA (mRNA) abundance in the pLVX-*RBP4* group (Figure 2c). Western blotting results showed that the RBP4 protein was upregulated in the pLVX-*RBP4* group (Figure 2d).

### 3.2. Transcriptome Profiling Analysis

High-throughput sequencing was performed in biological triplicates to elucidate the regulatory mechanism of response of GCs to *RBP4* overexpression. Six libraries of the pLVX-*RBP4* and control (CTRL) groups of GCs were sequenced. Table S2 summarizes the results on the six libraries. In this study, 4.505–5.011 Gb of raw bases were obtained, and 33.747–40.089 Mb of raw reads were generated from the three libraries of the pLVX-*RBP4* group. After quality control, 30.104–35.282 Mb of clean reads were obtained. Furthermore, Q20, Q30, and total mapped ratio were calculated (Table S2).

### 3.3. Differential Expression of Messenger RNAs in Granulosa Cells (GCs)

**Figure 3:**
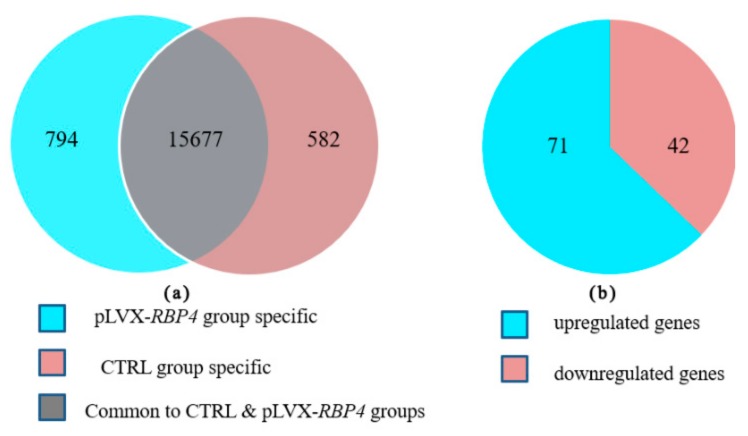
Sequencing analyses of genes expressed in GCs of pLVX-*RBP4* and CTRL groups. (**a**) Venn diagram showing the overlapping genes expressed in different groups. (**b**) The numbers of upregulated and downregulated differentially expressed genes (DEGs) under overexpression of retinol binding protein 4 (*RBP4*).

High-throughput sequencing was conducted in biological triplicates to identify differential expression of mRNAs in GCs. From 17,053 expressed mRNAs, 16,471 and 16,259 genes were expressed in the pLVX-*RBP4* and CTRL groups, respectively. Of these genes, 15,677 genes were expressed in both groups, whereas 794 and 582 genes were expressed only in the pLVX-*RBP4* and CTRL groups, respectively (Figure 3a; Table S3). A total of 113 differentially expressed genes (DEGs), including 71 upregulated genes and 42 downregulated genes, were identified in pLVX-RBP4 group compared with the CTRL group (Figure 3b; Table S4). The 10 most upregulated genes included *RBP4*, *HSPB1*, *MMP1*, *RBM34*, *S100A12*, *ITGA5*, *TOMM6*, *KIF20B*, *CCDC33*, and *MITD1*. The 10 most downregulated genes comprised *KCNMA1*, *IGFALS*, *LCN2*, *WIPF3*, *CD248*, *CRTC3*, *MAP1B*, *RAB3B*, *CPAMD8*, and *PRRC2C* (Table 1).

### 3.4. Validation of Differentially Expressed Messenger RNAs

**Figure 4:**
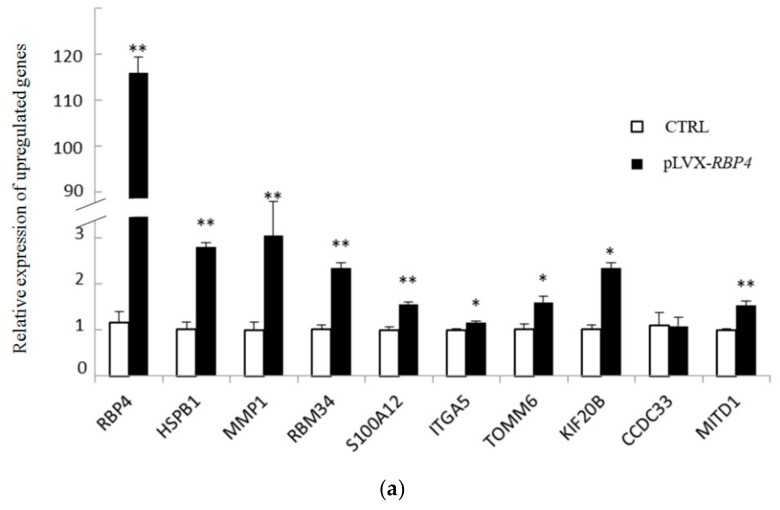

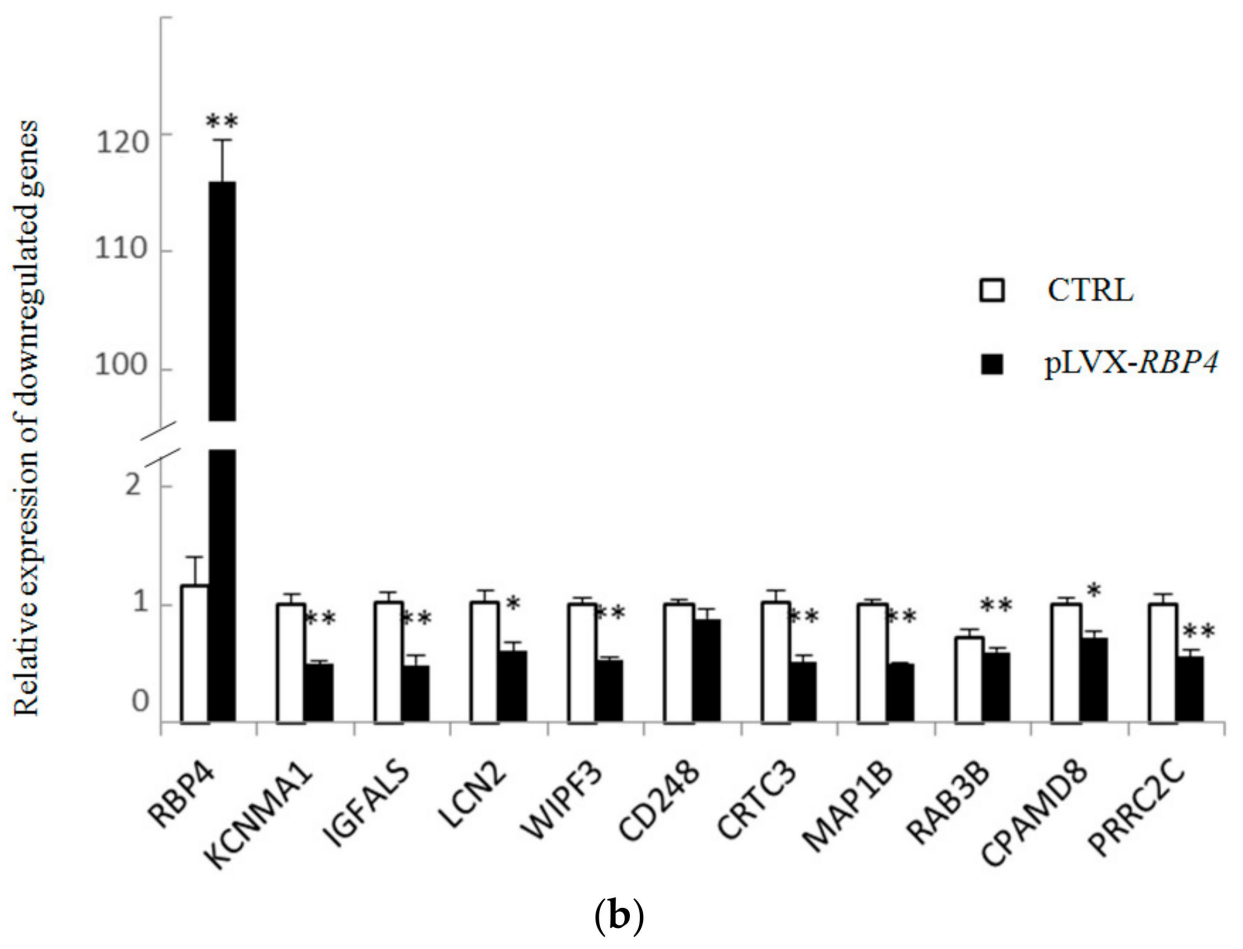
Relative expression of selected mRNAs was quantified by real-time quantitative polymerase chain reaction (RT-qPCR) (* *p* < 0.05; ** *p* < 0.01). (**a**) Relative expression of 10 upregulated genes. (**b**) Relative expression of 10 downregulated genes.

In accordance with previous reports, the top 10 up- and downregulated DEGs were selected for further validation by RT-qPCR analysis to validate the DEGs that were identified by RNA sequencing. We observed the upregulation of nine genes (*RBP4*, *HSPB1*, *MMP1*, *RBM34*, *S100A12*, *ITGA5*, *TOMM6*, *KIF20B*, and *MITD1*) by high-throughput sequencing, and this finding was confirmed by RT-qPCR. RT-qPCR also confirmed the downregulation of nine genes revealed by high-throughput sequencing. RNA sequencing showed that *CCDC33* and *CD248* were differentially expressed compared with the CTRL group, but RT-qPCR failed to confirm the data. The results demonstrated that differential expression was validated for 18 of 20 genes (Figure 4).

### 3.5. Gene Ontology Annotation and Kyoto Encyclopedia of Genes and Genomes Enrichment Analysis of Differentially Expressed Genes

Gene ontology includes biological processes (BP), cellular components (CC), and molecular functions (MF). GO terms with *p* < 0.05 were considered significantly enriched. Table 2 lists the top 30 significant GO terms, including BP, MF, and CC. Accordingly, the MF functions mainly related to protein binding, protein homodimerization activity, and growth factor binding were significantly enriched, including ribosomal subunit and mitochondrial part as GO terms for CC. In the BP category, the DEGs enriched in GO terms included protein folding, regulation of interleukin-1 production, mitotic cell cycle, and regulation of intrinsic apoptotic signaling pathway (Table 2).

To better illustrate their biological functions, we divided the DEGs into two groups: upregulated and downregulated. In the BP analysis, the enriched GO terms for upregulated DEGs converged on protein folding, regulation of interleukin-1 production, and interleukin-1 production. On the other hand, the downregulated DEGs were focused on exocytosis and cell migration (Figures S1 and S2). In the MF category, the upregulated DEGs were mainly enriched in protein binding and downregulated DEGs were enriched in insulin-like growth factor binding and growth factor binding (Figures S3 and S4). In the CC category, the upregulated DEGs were mainly enriched in protein binding involved in large ribosomal subunit, ribosomal subunit, and mitochondrion (Figure S5).

**Figure 5:**
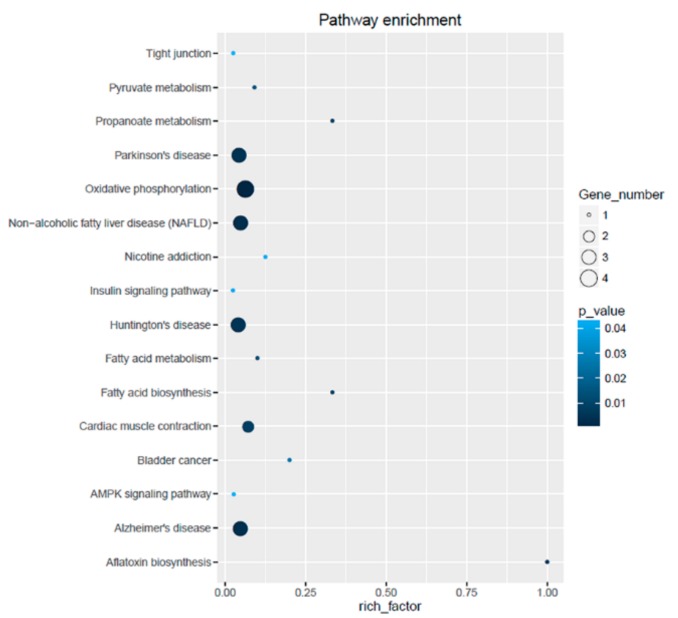
Detailed image of KEGG pathway enrichment.

All the DEGs were mapped to the KEGG database to further investigate their functions. A total of 28 DEGs were categorized into 16 pathways (Figure 5). The DEGs were determined to be involved in key pathways, such as oxidative phosphorylation, Parkinson’s disease, non-alcoholic fatty liver disease (NAFLD), Huntington’s disease, cardiac muscle contraction, Alzheimer’s disease, fatty acid biosynthesis, AMPK signaling pathway, insulin signaling pathway, and tight junction. Most of these pathways are related to metabolic diseases.

### 3.6. Protein–Protein Interaction (PPI) Network Analysis

**Figure 6:**
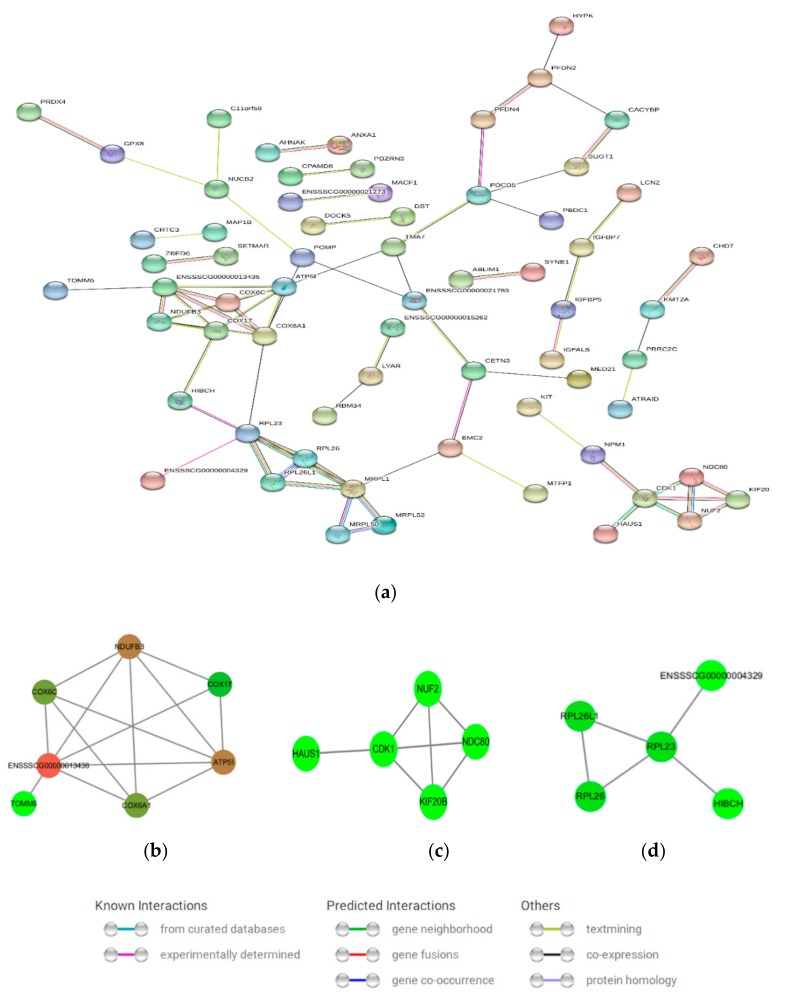
Protein–protein interaction (PPI) of the DEGs. (**a**) An integral PPI network, (**b**) Oxidative phosphorylation network, (**c**) Cell cycle network, (**d**) Cell ribosome network. The nodes represent proteins. The node color represents the connectivity between genes. Deeper color indicates a better connectivity between adjacent genes.

An integral PPI network (Figure 6a) and three sub-networks (Figure 6b–d) were constructed by mapping the upregulated and downregulated DEGs. The integral network consisted of 65 proteins. Figure 6b contained seven proteins (COX17, cytochrome oxidase subunit 6A1 (COX6A1), COX6C, ATP5I, ubiquinone oxidoreductase subunit B3 (NDUFB3), TOMM6, and ENSSSCG00000013436), which were mainly related to oxidative phosphorylation, NAFLD, and Alzheimer’s disease. Figure 6c–d were related to cell cycle and cell ribosome, respectively. Figure 6d consisted of RPL26, RPL26L1, and RPL23, which are cytoplasmic ribosomal proteins. 

## 4. Discussion

Granulosa cells are important somatic cells that surround oocytes. These cells are involved in ovarian follicular development in pigs. RBP4 is an approximately 21 KD peptide that acts as a carrier of vitamin A in the plasma. This protein is one of the identified adipokines that are associated with IR, obesity, and type 2 diabetes [9]. RBP4 is highly related to ovarian diseases, such as PCOS, ovarian cancer in humans [12,33,34,35,36], and ovarian cysts in swine [22], which may be caused by endocrine disorders. Furthermore, previous studies have shown that RBP4 could promote cell proliferation. A study by Li indicated that RBP4 could increase the proliferation and invasion of HTR8 cells through suppressing PI3K/AKT signaling [37]. Another study demonstrated that *RBP4* affected proliferation, differentiation, and mineralization of MC3T3-E1 cell line in vitro [38]. 

In the present study, we identified the effects of *RBP4* on GCs by transcriptomic analysis following *RBP4* overexpression. RNA-based overexpression of *RBP4* changed the expression levels of 113 DEGs. In addition, our data revealed the effects of *RBP4* of DEGs that are important to pathways in GCs, including oxidative phosphorylation, NAFLD, Alzheimer’s disease, fatty acid biosynthesis, AMPK signaling pathway, and insulin signaling pathway.

Oxidative phosphorylation is involved in GC apoptosis and follicular atresia [39]. Abnormal mitochondrial oxidative phosphorylation occurs in obese girls with type 2 diabetes and IR, which are related to RBP4 [40]. The insulin signaling pathway participates in follicular development, GC proliferation, and developmental ability of oocytes in vitro [41,42]. Nevertheless, insulin pathway is also associated with ovarian diseases, such as PCOS, abnormal steroidogenesis, and ovarian cancer [43,44]. In mammals, the AMPK signaling pathway is involved in the regulation of many cellular functions, and studies indicated that AMPK signaling play roles in bovine GCs to express a proliferative and steroidogenic phenotype [45]. Another study recently proposed a relationship between the GZYKF (a Chinese medicine formula) effect and AMPK pathway that may underlie the modulation of GC autophagy [46].

The significant role of RBP4 is the transport of retinol from the liver to peripheral tissues. A recent study found that RBP4 was located in the granulosa and inner theca cell layers and the expression level of *RBP4* in ovarian follicles increased with follicular sizes. GCs treated with increasing FSH or LH promoted the expression of *RBP4* [24]. However, the minor number of DEGs was detected in this article. Further study is needed to clarify if RBP4 exerts its biological function through the synergy of other factors, such as transhyretin, FSH, LH, and cell membrane receptor STRA6. KEGG results are not directly related to reproductive pathways, nevertheless, oxidative phosphorylation, fatty acid biosynthesis, and AMPK signaling can affect the physiological function of GCs. Available data focusing on the relationship of *RBP4* and type 2 diabetes mellitus, PCOS, and ovarian cancer have been originated from population-based prospective studies. However, in present study, some attractive DEGs related to reproduction and cell cycle were detected, such as *MMP1*, IGF signaling related genes (*IGFBP5*, *IGFBP7*, and *IGFALS*), *HSPB1*, and cell-cycle gene (*CDK1*).

Li et al. observed that *RBP4* knockdown decreased cell invasion, whereas *RBP4* overexpression increased the invasion of HTR8/SVneo cells. Moreover, the production of *MMPs* increased with the increase in *RBP4* expression [37]. The *RBP4*-stimulated expression of *MMP1* in the present study supports the above notion. Furthermore, our results confirmed for the first time that *IGFBP5*, *IGFBP7*, and *IGFALS* were detected as DEGs in GCs overexpressing *RBP4*. Among these DEGs, *IGFBP5* and *IGFALS* were downregulated, and *IGFBP7* was upregulated. Interestingly, *IGFBP5* and *IGFALS* were downregulated simultaneously, as IGF1, IGFBP5, and IGFALS function in a form of ternary complex for IGF signaling, which has been shown to play important roles in a variety of physiological processes, including protein chaperoning, steroidogenesis, and protection against apoptosis [47,48,49,50]. Upregulation of the expression of *HSPB1* by *RBP4* may have significant consequences for the production of *HSPB1* in GCs. HSPB1 is a protein that appears to antagonize a molecular pathway for stress [51], and HSPB1 was demonstrated to be associated with folliculogenesis and steroidogenesis in bovine ovary [52]. Cyclin-dependent kinase 1 (*CDK1*) is a key regulator of the cell cycle and RNA transcription [53]. The hubs of *CDK1* are upregulated in bovine GCs in luteal phase and small healthy follicles [54]. Meanwhile, in this research, the expression of *CDK1*, which also acts as a hub of PPI, was upregulated in GCs challenged with *RBP4*. In addition, previous studies have shown that *RBP4* could promote cell proliferation. These results suggest that overexpression of *RBP4* promoted cell cycle in porcine GCs. Our data indicates that RBP4 plays a potential role in in ovarian folliculogenesis and pathogenesis.

## 5. Conclusions

In this study, we compared the differential expression of genes of porcine GCs overexpressing *RBP4* by using high-throughput sequencing. In addition, our transcriptomic data support that several genes are involved in important biological processes associated with folliculogenesis and pathogenesis. As such, these findings provide novel insights on the role of *RBP4* in porcine GCs.

## Figures and Tables

**Table 1:** Top 10 upregulated and downregulated genes in granulosa cells (GCs) of pLVX-*RBP4* group compared with the CTRL group.

Gene Symbol	Gene Name	Description and Summary of the Function
Upregulated		
*RBP4*	Plasma retinol binding protein 4	Plays a role in retinol transport; involved in insulin resistance and diabetes
*HSPB1*	Heat shock protein 27	Provides thermotolerance in vivo and support cell survival under stress conditions
*MMP1*	Matrix metallopeptidase 1	Involved in the breakdown of extracellular matrix
*RBM34*	RNA binding motif protein 34	Ubiquitous expression in testis, lymph node, and other tissues
*S100A12*	S100 calcium-binding protein A12	Involved in the regulation of cell cycle progression and differentiation and specific calcium-dependent signal transduction
*ITGA5*	Integrin α-5	Participates in cell-surface mediated signaling
*TOMM6*	Mitochondrial import receptor subunit TOM6	May play a role in breast carcinogenesis
*KIF20B*	kinesin-like protein 20A	Could promote cancer progression and is essential for cytokinesis in cell cycle
*CCDC33*	Coiled-coil domain-containing protein 33	May be regulated by hormone and nutrition
*MITD1*	Microtubule interacting and trafficking domain containing 1	Participates in the abscission phase of cytokinesis
Downregulated		
*KCNMA1*	Potassium calcium-activated channel subfamily M α 1	Relates with several cancers, such as breast carcinoma, prostate cancer, and cervical cancers
*IGFALS*	Insulin like growth factor binding protein acid labile subunit	Act as IGFBPs, binding IGFs exerts its function
*LCN2*	Lipocalin-2	Estradiol can regulate the expression of *LCN2* and is linked to IR
*WIPF3*	Wasl interacting protein family member 3	May be a regulator of cytoskeletal organization and play a role in spermatogenesis
*CD248*	Endosialin	Essential for embryo development
*CRTC3*	Creb-regulated transcription coactivator 3	Transcriptional coactivator; plays roles in adipose development and energy metabolism
*MAP1B*	Microtubule-associated protein 1B	Plays an important role in development and function of the nervous system
*RAB3B*	Ras-related protein-3B	May play a role in regulating GLUT4 translocation in adipocytes
*CPAMD8*	C3 and pzp-like α-2-macroglobulin domain-containing protein 8	May be associated with cataract development
*PRRC2C*	Proline rich coiled-coil 2C	Involved in the development of basement membrane

**Table 2:** Top 30 significant terms from Enrich Gene Ontology for biological processes (BP), molecular functions (MF), and cellular components (CC).

GO Terms	Gene Name	Gene Ratio	*p*-Value
Biological process (BP)			
GO:0006457-protein folding	*HSPB1, PFDN4, PFDN2, PRDX4*	6.3%	0.000427
GO:0006887-exocytosis	*KIT, RAB3B*	2.7%	0.00471
GO:0032652-regulation of interleukin-1 production	*HSPB1, ANXA1*	10.5%	0.00508
GO:0016477-cell migration	*KIT, PEAK1, CD248*	1.1%	0.00512
GO:0032612-interleukin-1 production	*HSPB1, ANXA1*	10.0%	0.00562
GO:0048870-cell motility	*KIT, PEAK1, CD248*	1.0%	0.00688
GO:0051674-localization of cell	*KIT, PEAK1, CD248*	1.0%	0.00688
GO:0040011-locomotion	*KIT, PEAK1, CD248*	0.9%	0.0107
GO:0006928-movement of cell or subcellular component	*KIT, PEAK1, CD248*	0.9%	0.0117
GO:0000278-mitotic cell cycle	*MITD1, CDK1, NUF2, RPL26*	6.3%	0.0127
Molecular function (MF)			
GO:0005520-insulin-like growth factor binding	*IGFBP5, IGFALS*	12.5%	0.000330
GO:0044183-protein binding involved in protein folding	*HSPB1, PFDN2*	20.0%	0.00137
GO:0042803-protein homodimerization activity	*HSPB1, MITD1, CACYBP, AIMP1, PRDX4*	3.3%	0.00148
GO:0003735-structural constituent of ribosome	*MRPL52, RPL26, RPL26L1, RPL23*	4.4%	0.00155
GO:0019838-growth factor binding	*IGFBP5, IGFALS*	5.7%	0.00161
GO:0004713-protein tyrosine kinase activity	*KIT, PEAK1*	4.3%	0.00277
GO:0016684-oxidoreductase activity, acting on peroxide as acceptor	*GPX8, PRDX4*	9.1%	0.00675
GO:0016209-antioxidant activity	*GPX8, PRDX4*	5.7%	0.0166
GO: 0051082-unfolded protein binding	*PFDN4, PFDN2*	5.7%	0.0166
GO:0046983-protein dimerization activity	*HSPB1, MITD1, CACYBP, AIMP1, PRDX4*	1.9%	0.0169
Cellular component (CC)			
GO:0015934-large ribosomal subunit	*MRPL52, RPL26, RPL26L1, RPL23*	7.8%	0.000231
GO:0044391-ribosomal subunit	*MRPL52, RPL26, RPL26L1, RPL23*	4.9%	0.00135
GO:0005739-mitochondrion	*MRPL50, NDUFB3, COX17, MTFP1, MRPL52, COX6C, ATP5I, PFDN2, PRDX4*	1.9%	0.00141
GO:0005840-ribosome	*MRPL52, RPL26, RPL26L1, RPL23*	3.9%	0.00327
GO:0044429-mitochondrial part	*NDUFB3, COX17, MTFP1, MRPL52, COX6C, ATP5I*	2.2%	0.00490
GO:0031967-organelle envelope	*NDUFB3, CACYBP, COX17, MTFP1, COX6C, ATP5I*	2.2%	0.00567
GO:0031975-envelope	*NDUFB3, CACYBP, COX17, MTFP1, COX6C, ATP5I*	2.2%	0.00567
GO: 0005740-mitochondrial envelope	*NDUFB3, COX17, MTFP1, COX6C, ATP5I*	2.5%	0.00660
GO: 0031970-organelle envelope lumen	*CACYBP, COX17*	9.5%	0.00700
GO:0005743-mitochondrial inner membrane	*NDUFB3, MTFP1, COX6C, ATP5I*	3.1%	0.00749

## Supplementary Materials

The following are available online at https://www.mdpi.com/2073-4425/10/8/615/s1. Table S1: Primers of RT-qPCR used in the present study; Table S2: Sequencing quality statistics for the GCs of pLVX-RBP4 and CTRL groups; Table S3: Genes expressed in pLVX-RBP4 group and CTRL group. Table S4: DEGs of pLVX-RBP4 group compared to CTRL group in the GCs. Figure S1: BP analysis of downregulated DEGs; Figure S2: BP analysis of upregulated DEGs; Figure S3: MF analysis of downregulated DEGs; Figure S4: MF analysis of upregulated DEGs; Figure S5: CC function analysis of upregulated DEGs.
