# Supplementary material for: Transcriptomic Analysis of Porcine Granulosa Cells Overexpressing Retinol Binding Protein 4
Source: Genes (Basel). 2019 Aug 13;10(8):615. doi: 10.3390/genes10080615 (PMC6722559; doi:10.3390/genes10080615)
Supplement: Supplementary file 1 [file genes-10-00615-s001.zip › supplementary/Table S1Primers of RT-qPCR used in the present study.docx]

Table S1 Primers of RT-qPCR used in the present study

| Name | Primer sequence(5’-3’) | Length(bp) |
| --- | --- | --- |
| GAPDH | F: TGGGCATGAACCATGAGAAG  R: CCACGATGCCGAAGTTGTC | 117 |
| RBP4 | F: GGGCGTAGCATCCTTCCT  R: TCCGATTTGCCATCACAG | 264 |
| RBM34 | F: TTGTGGCTGTAAGGATCGTG  R: TTCTCAAAGAGCACGTAGCC | 73 |
| S100A12 | F: ACACCCTCAAGAACACCAAAG  R: TTGTCGTGGGCAGTTATCAG | 136 |
| CPAMD8 | F: GTATGTGTTGCCGAAGTTTGAG  R: CATGTTGATCGTTAGAGTCCCC | 136 |
| PRRC2C | F: AAGTCCAAATAGCCAGTCCAG  R: TGTCATTCTCACAGTGGCAG | 85 |
| MITD1 | F: GAGTCACGGTATCCACAAGC  R: TGTTTCATCTTTGGTGCCTTTC | 87 |
| CCDC33 | F: GAAAGCCAGTTAGAGGACTCAG  R: TGATGGTGTTGGAAGGGTG | 109 |
| KIF20B | F: TCTCCAGTAACGCACAGAAAG  R: GTTCAGTTTCAACCTTGCCAG | 141 |
| TOMM6 | F: TCGTCAAATGAAGCTCCCG  R: AGTCATTCCTATCGGTAGCAAAG | 85 |
| IGFBP5 | F: AAGAAGCTGACCCAGTCCAAG  R: GACCCTGCTCAGATTCCTGT | 100 |
| KIT | F: GATGAGTTGGCCCTAGACCTG  R: TGCCATCCACTTCACGGGTA | 234 |
| ITGA5 | F: GGGTCCTTTGGTGTGGACAA  R: CGCTCCTCTGGGTTGAACAT | 104 |
| MMP1 | F: ACTTGTATCGTGTGGCTGCT  R: GGGACAGCTGAACATCACCA | 117 |
| KCNMA1 | F: GCACCCAAGGAGATAGAGAAAG  R: TCGCCAAAGATACAGACCAC | 92 |
| IGFALS | F: TCAATGACAACCAGATCCAGG  R: TGCCAGACAAGTTCATGACG | 81 |
| LCN2 | F: AGGCCGGTTTAAGATGTACAC  R: ATCCAGTTGTCACAGAGCTG | 102 |
| WIPF3 | F: ACGAGTATAAGCCATGCCAG  R: GCGTTTCTGCCTTTGATGTC | 121 |
| RAB3B | F: CCTTCCTCTTCCGCTATGC  R: ACCTTAAAGTCGATGCCCAC | 73 |
| CD248 | F: CCATGTGAGCAGCAGTGTGA  R: GATCTGGCACTCATCCGTGT | 120 |
| HSPB1 | F：TCCCTGGACGTCAACCACTT  R：AAACACCGGGAAATGAAGCC | 122 |
