# Supplementary material for: Transcriptomic Analysis of Porcine Granulosa Cells Overexpressing Retinol Binding Protein 4
Source: Genes (Basel). 2019 Aug 13;10(8):615. doi: 10.3390/genes10080615 (PMC6722559; doi:10.3390/genes10080615)
Supplement: Supplementary file 1 [file genes-10-00615-s001.zip › supplementary/Table S2 Sequencing quality statistics for the GCs of CTRL group and pLVX-RBP4 group.docx]

TableS2. Sequencing quality statistics for the GCs of CTRL group and pLVX-*RBP4* group

| Index | CTRL-1 | CTRL -2 | CTRL -3 | pLVX-RBP4-1 | pLVX-RBP4-2 | pLVX-RBP4-3 |
| --- | --- | --- | --- | --- | --- | --- |
| Raw Reads(M) | 32.565 | 29.244 | 31.207 | 40.083 | 33.747 | 40.089 |
| Raw Bases(G) | 4.268 | 3.840 | 3.900 | 5.010 | 4.505 | 5.011 |
| Clean Reads(M) | 28.777 | 25.758 | 27.038 | 35.282 | 30.104 | 34.995 |
| Clean Bases(G) | 3.639 | 3.261 | 3.254 | 4.259 | 3.893 | 4.218 |
| Clean Q20(G) | 3.548(97.5%) | 3.180(97.5%) | 3.159(97.1%) | 4.137(97.2%) | 3.806(97.8%) | 4.090(97.0%) |
| Clean Q30(G) | 3.412(93.7%) | 3.058(93.8%） | 3.030(93.1%） | 3.971(93.2%) | 3.669(94.2%） | 3.916(92.9%） |
| Total Reads(M) | 28.777 | 25.758 | 27.038 | 35.282 | 30.104 | 34.995 |
| Total Mapped(M) | 20.730(72.0%) | 19.095(74.1%) | 19.812(73.3%) | 27.192(77.1%) | 23.028(76.5%) | 27.149(77.6%) |
| Multiple Mapped(M) | 1.356(4.71%) | 1.268(4.92%) | 1.331(4.92%) | 1.797（5.09%） | 1.469(4.88%) | 1.801(5.15%) |
| Uniquely Mapped(M) | 19.374(67.32%) | 17.827(69.21%) | 18.480(68.35%) | 25.394(71.97%) | 21.559(71.62%) | 25.348(72.43%) |

Abbreviation: Q20, the percentage of bases with a Phred value >20; Q30, the percentage of bases with a Phred value >30
