# Supplementary figures and images for: Transcriptomic Analysis of Porcine Granulosa Cells Overexpressing Retinol Binding Protein 4
Source: Genes (Basel). 2019 Aug 13;10(8):615. doi: 10.3390/genes10080615 (PMC6722559; doi:10.3390/genes10080615)

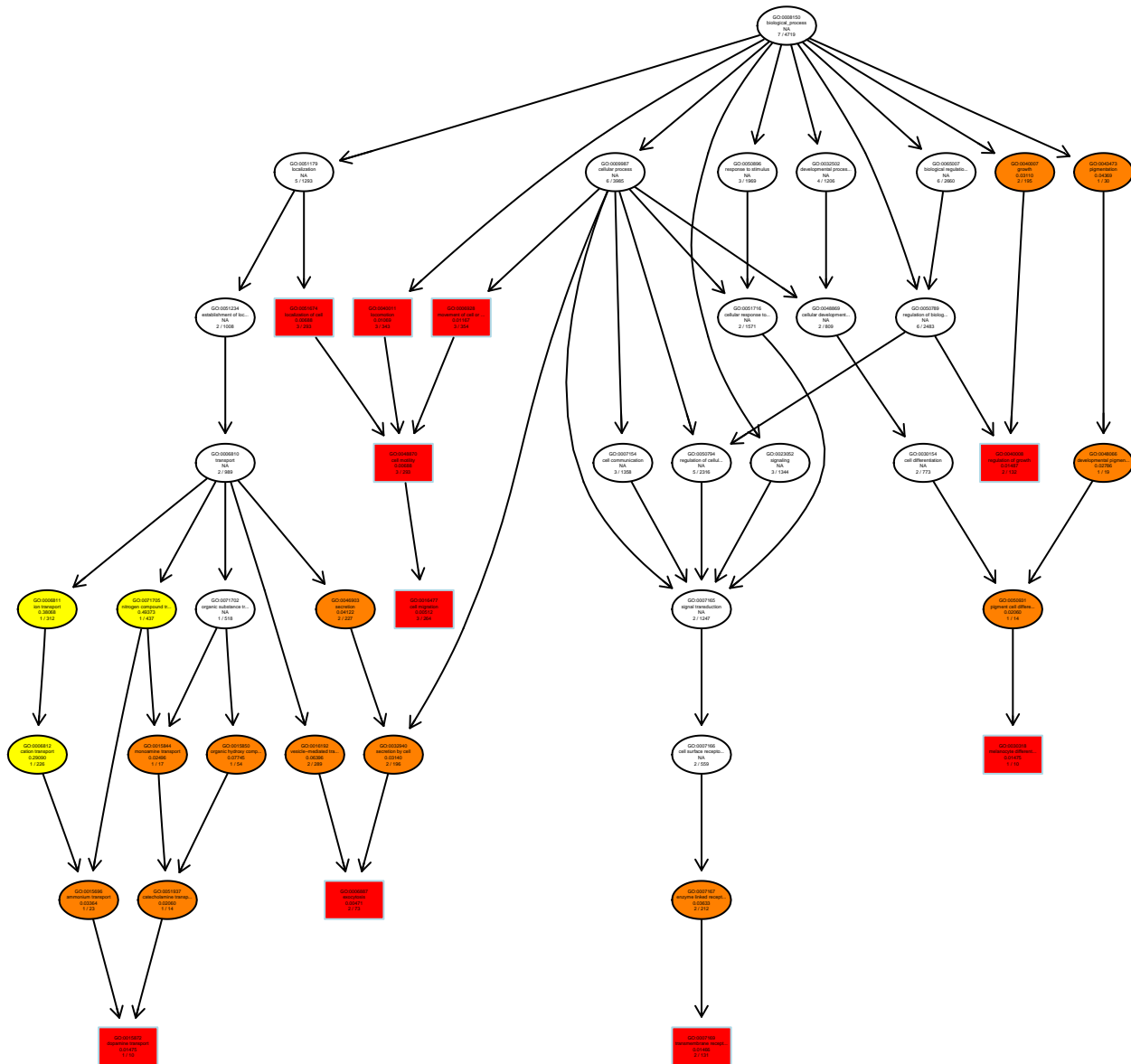

Supplement: Supplementary file 1 [file genes-10-00615-s001.zip › supplementary/Figure S1-down.go.bp.pdf]

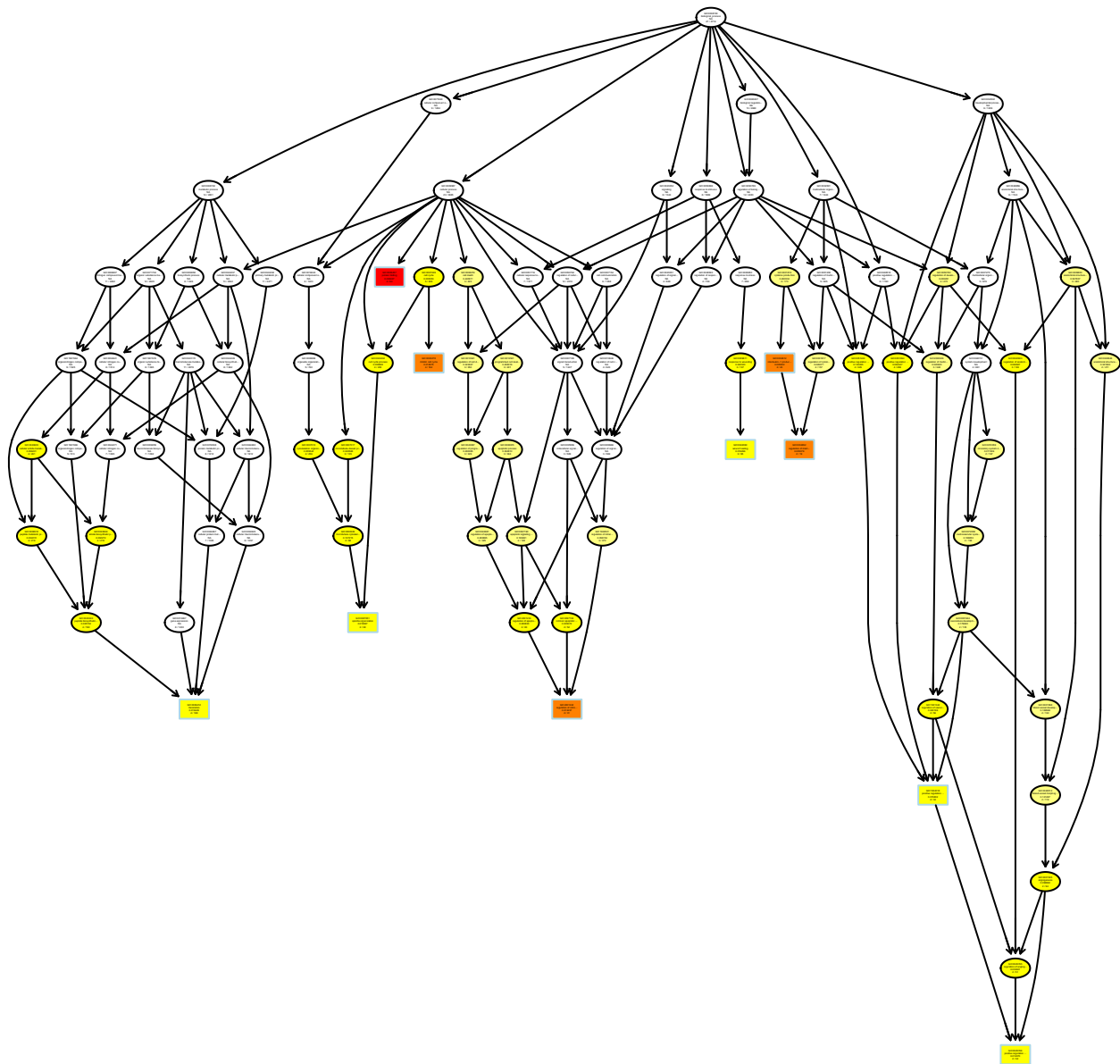

Supplement: Supplementary file 1 [file genes-10-00615-s001.zip › supplementary/Figure S2-up.go.bp.pdf]

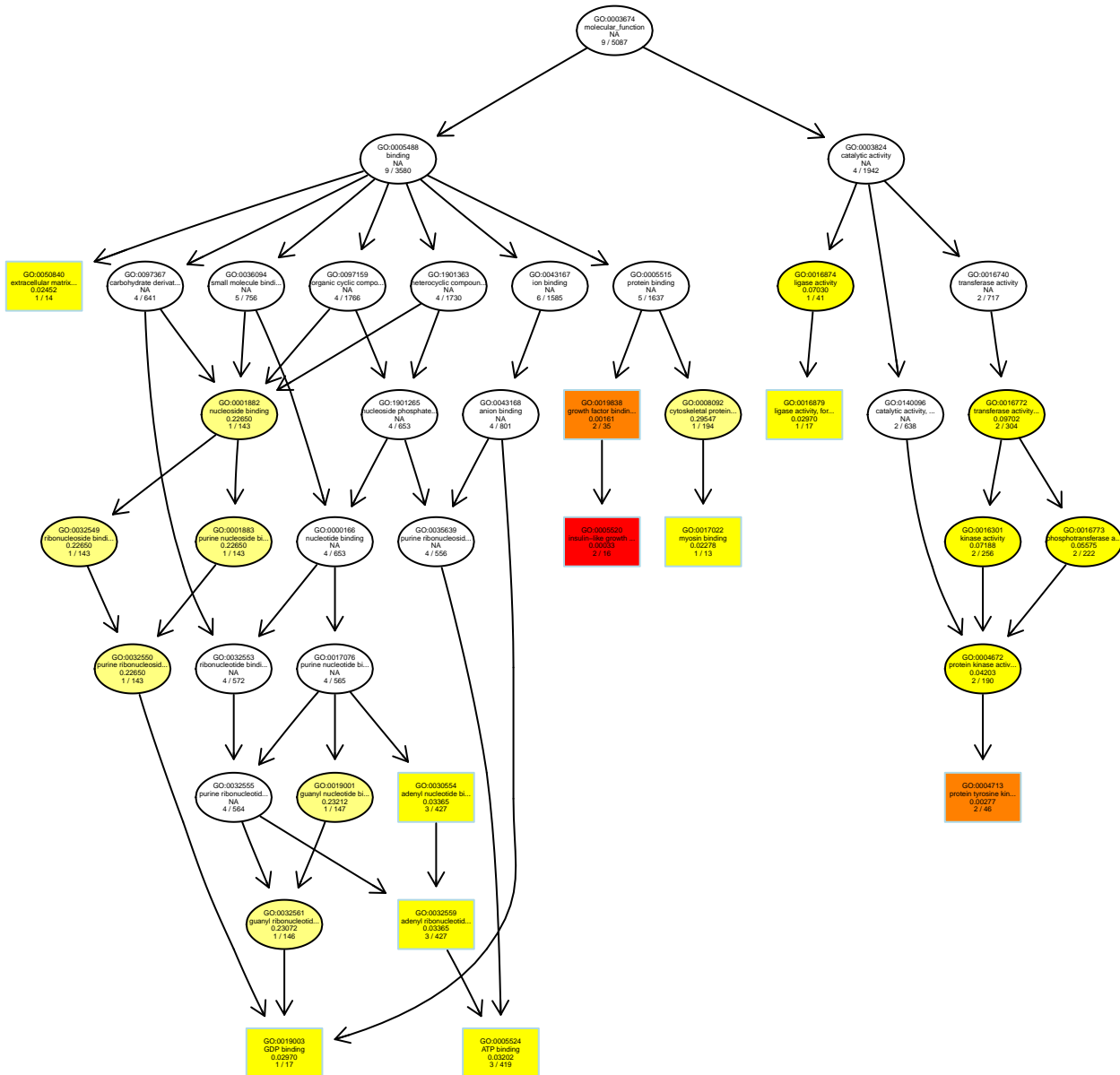

Supplement: Supplementary file 1 [file genes-10-00615-s001.zip › supplementary/Figure S3-down.go.mf.pdf]

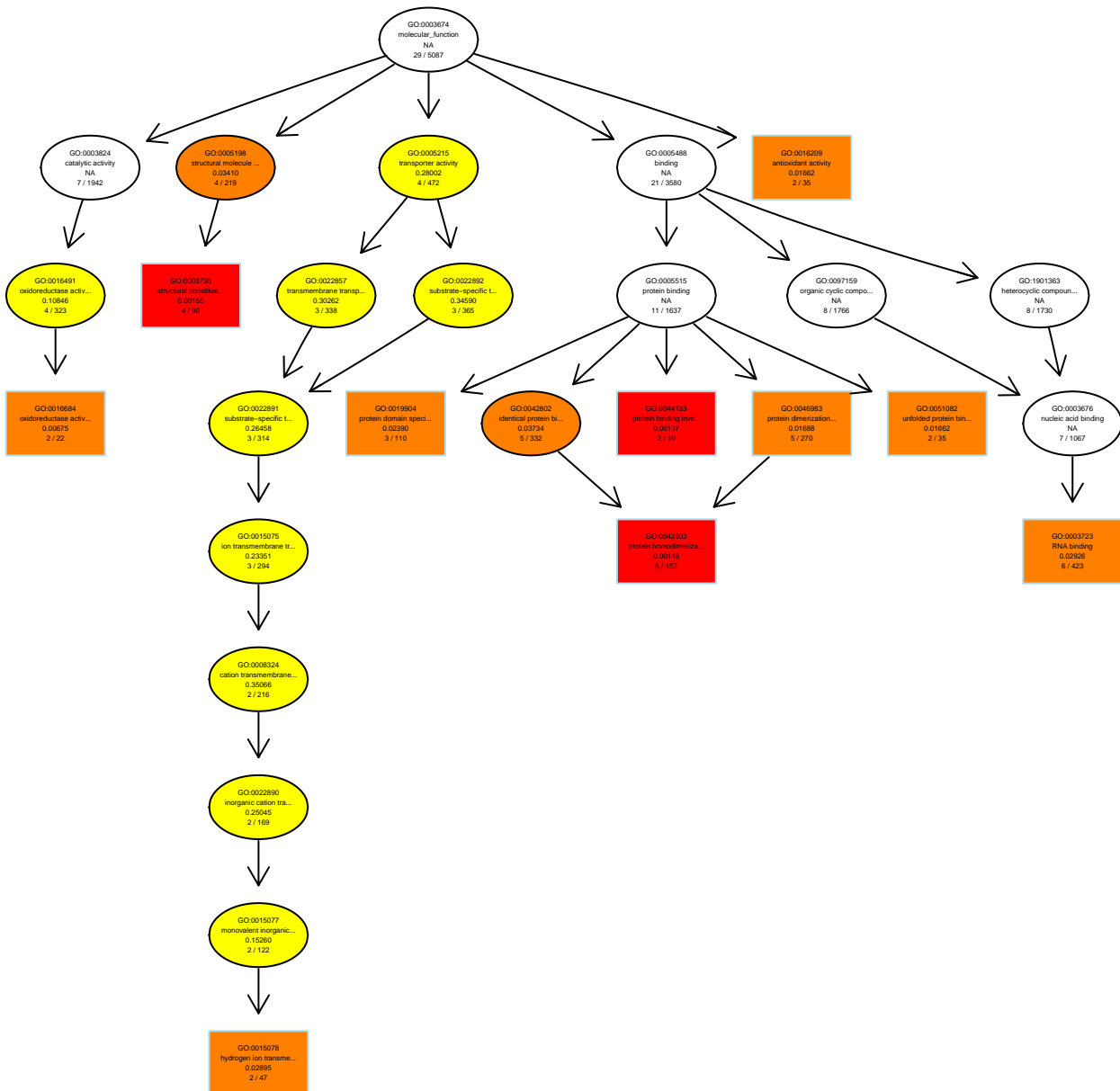

Supplement: Supplementary file 1 [file genes-10-00615-s001.zip › supplementary/Figure S4-up.go.mf.pdf]

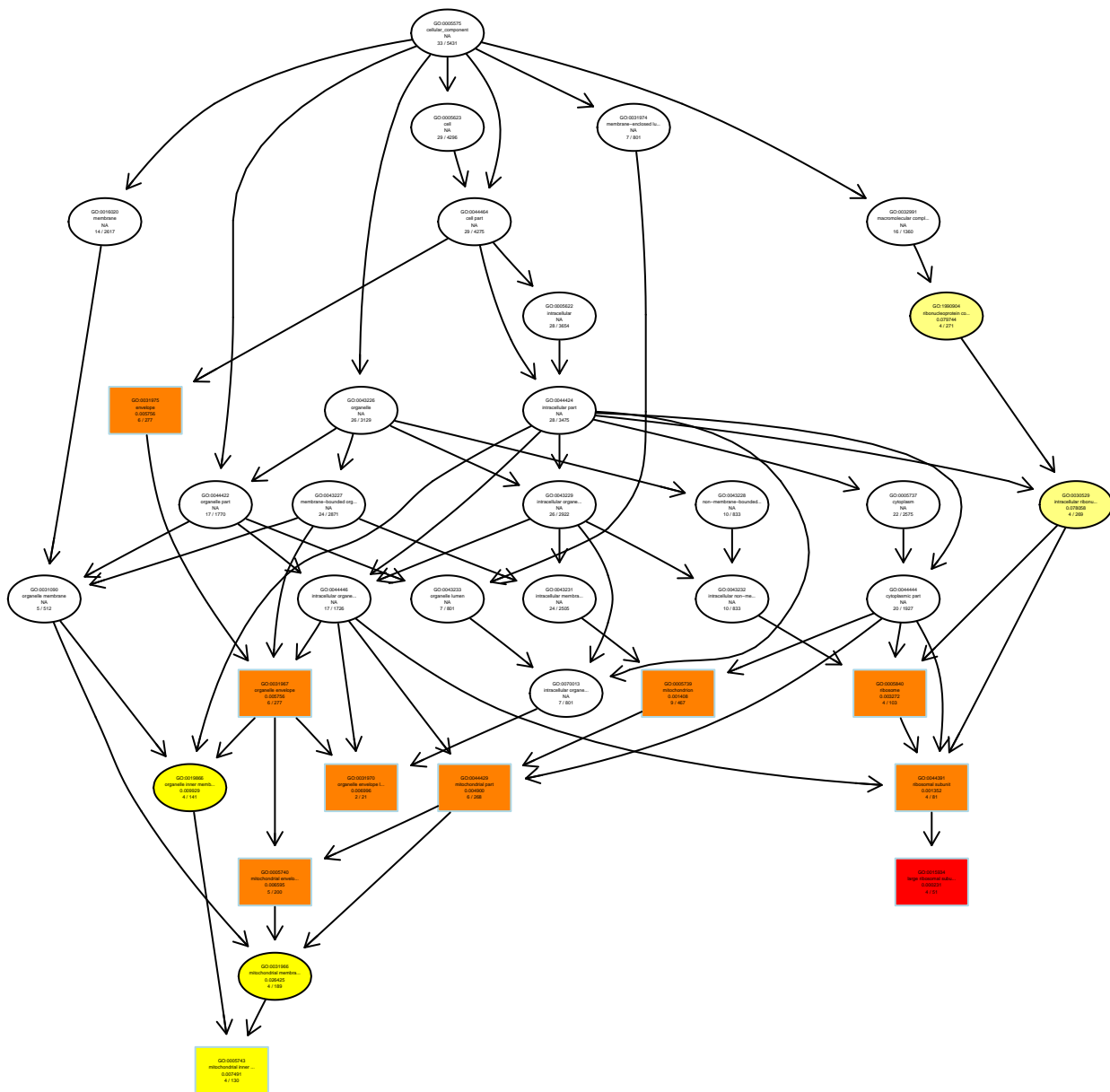

Supplement: Supplementary file 1 [file genes-10-00615-s001.zip › supplementary/Figure S5-up.go.cc.pdf]
